# Circular RNAs in inflammatory bowel disease: a review of mechanisms, biomarkers and therapeutic potential

**DOI:** 10.3389/fimmu.2025.1540768

**Published:** 2025-04-24

**Authors:** Le Yang, Huahui Li, Min Tang, Lingnan He, Lijun Yang

**Affiliations:** ^1^ Department of Gastroenterology, Yiyang Central Hospital, Yiyang, China; ^2^ Institute of Biomedical and Health Engineering, Chinese Academy of Sciences Shenzhen Institutes of Advanced Technology, Shenzhen, China; ^3^ Department of Pharmacy, Yiyang Medical College, Yiyang, China; ^4^ Endoscopy Center, Department of Gastroenterology, Shanghai East Hospital, School of Medicine, Tongji University, Shanghai, China

**Keywords:** circular RNA, inflammatory bowel disease, ulcerative colitis, Crohn’s disease, biomarker, diagnosis, prognosis

## Abstract

Inflammatory bowel disease (IBD) is an autoimmune disease of unknown etiology characterized by recurrent chronic inflammation of the gastrointestinal tract. The incidence of IBD is increasing and has become a focus of research on digestive diseases. Despite advances in understanding its multifactorial etiology, including genetic predisposition, microbiome dysbiosis, and immune dysregulation. However, the molecular mechanisms driving IBD pathogenesis remain incompletely elucidated. Circular RNA (circRNA) is a stable single-stranded RNA with a closed-loop structure and conserved nature. circRNA possesses multiple functions, such as adsorption of microRNAs and RNA-binding proteins, and is involved in the regulation of gene splicing and transcription, as well as protein translation. However, circRNAs in IBD progression and their clinical potential as biomarkers or therapeutic targets are yet to be systematically explored. In this review, we comprehensively synthesize recent advancements in circRNA research related to IBD, integrating evidence from *in vitro*, *in vivo*, and clinical studies. We systematically analyze aberrant circRNA expression profiles in IBD tissues (e.g., intestinal mucosa, peripheral blood, and exosomes) and discuss their mechanism of action contributions to inflammation, intestinal epithelial barrier dysfunction, autophagy, intestinal fibrosis, and colitis-associated cancer (CAC). Furthermore, we evaluate methodologies for circRNA detection and therapeutic modulation, including RNA interference, viral vector delivery, and PLGA MSs delivery system strategies. This review highlights the potential of circRNA-focused strategies in the diagnosis and treatment of IBD, offering a scientific foundation for advancing precision medicine in IBD management.

## 1 Introduction

Inflammatory bowel disease (IBD), which includes ulcerative colitis (UC) and Crohn’s disease (CD), is a chronic intestinal inflammatory disorder of unknown etiology. The incidence of IBD is rising globally, making it a significant health concern (1). The etiology of IBD is primarily associated with genetics, environmental factors, dysbiosis of the gut microbiome, and immune system disorders, leading to dysregulation of intestinal barriers and immune functions (2, 3). The diagnosis of IBD relies on clinical assessment, laboratory tests, imaging, endoscopy, and histological examination. However, there is currently no definitive diagnostic criterion. Identifying new therapeutic targets and diagnosis biomarkers is crucial for early detection and personalized treatment.

Non-coding RNAs, such as microRNAs(miRNA), long non-coding RNAs(lncRNA), and circRNA, do not encode proteins but play a crucial role in regulating gene expression within cellular processes (4). Early studies focused on miRNAs and lncRNAs revealed their critical roles in modulating IBD-associated pathways—such as NF-κB activation, immune homeostasis, and intestinal barrier integrity (5–7). CircRNAs are a class of covalently closed circular RNA molecules that lack a 5’ cap and a 3’ poly(A) tail (8). This structural feature confers exceptional stability and resistance to exonuclease-mediated degradation, enabling circRNAs to be stably expressed in the cytoplasm or stored within exosomes compared to their linear counterparts (9). They are widely expressed in mammalian tissues and cells, exhibiting tissue and cell type-specific patterns (10), and are involved in various physiological and pathological processes (11). They exhibit aberrant expression in various diseases, including (12, 13), cardiovascular diseases (14), autoimmune diseases (15), neurological disorders (16), and aging processes (17). CircRNAs can be detected in multiple body fluids (peripheral blood, saliva, urine, semen, etc.) and tissues/cells (18). The unique characteristics of circRNAs make them potential biomarkers and therapeutic targets for the diagnosis and treatment of human diseases (19, 20).

The role and molecular mechanism of circRNAs in IBD remain incompletely understood. Although circRNAs in IBD have not been extensively studied, emerging evidence has identified differential expression of specific circRNAs(e.g., circRNA_103516,circ_004662) between IBD patients and healthy controls, with these molecules playing pivotal roles in disease pathogenesis (21, 22). Accumulating data further suggests their potential clinical utility in IBD diagnosis, prognosis, and therapeutic interventions (7, 23). Therefore, the functional contributions and pathogenic mechanisms of circRNAs in IBD urgently require further investigation to achieve a comprehensive understanding.

Recent reviews, such as the comprehensive work by Lun et al, have systematically summarized the roles of circRNAs in IBD pathogenesis and immune regulation (24). However, emerging studies over the past years have revealed novel mechanistic insights into circRNA-mediated intestinal barrier dysfunction, autophagy modulation, intestinal fibrosis progression, and Colitis-associated cancer(CAC). Building on these findings, this review systematically synthesizes cutting-edge research advances to address critical questions regarding the roles of circRNAs in IBD. Our work provides principal contributions: Firstly, we elucidated the dynamic differential expression profile of circular RNAs (circRNAs) in inflammatory bowel disease (IBD), with validation confirming their potential as non-invasive biomarkers in plasma exosomes (e.g., circ103124). Secondly, we explored the mechanistic role of circRNAs in IBD in detail. Regarding their function as microRNA (miRNA) sponges, we discussed issues such as inflammation amplification, immune dysregulation, intestinal mucosal barrier disruption, and autophagy modulation. Concerning RNA-binding proteins (RBPs), we focused on post-transcriptional regulation, particularly in intestinal mucosal repair and autophagy regulation. Furthermore, in the field of epigenetics, we investigated the role of N6-methyladenosine(m6A)-dependent epigenetic mechanisms in IBD. Additionally, we introduced the circRNA-mediated regulatory network involved in intestinal fibrosis and colitis-associated cancer (CAC).Finally, we evaluated innovative therapeutic delivery systems, specifically the application of poly(lactic-co-glycolic acid) (PLGA) microspheres for circRNA-targeted interventions in IBD, aiming to provide a more effective and safer therapeutic strategy for patients with inflammatory bowel disease.

## 2 Aberrant expression of CircRNAs in IBD

**Figure 1:**
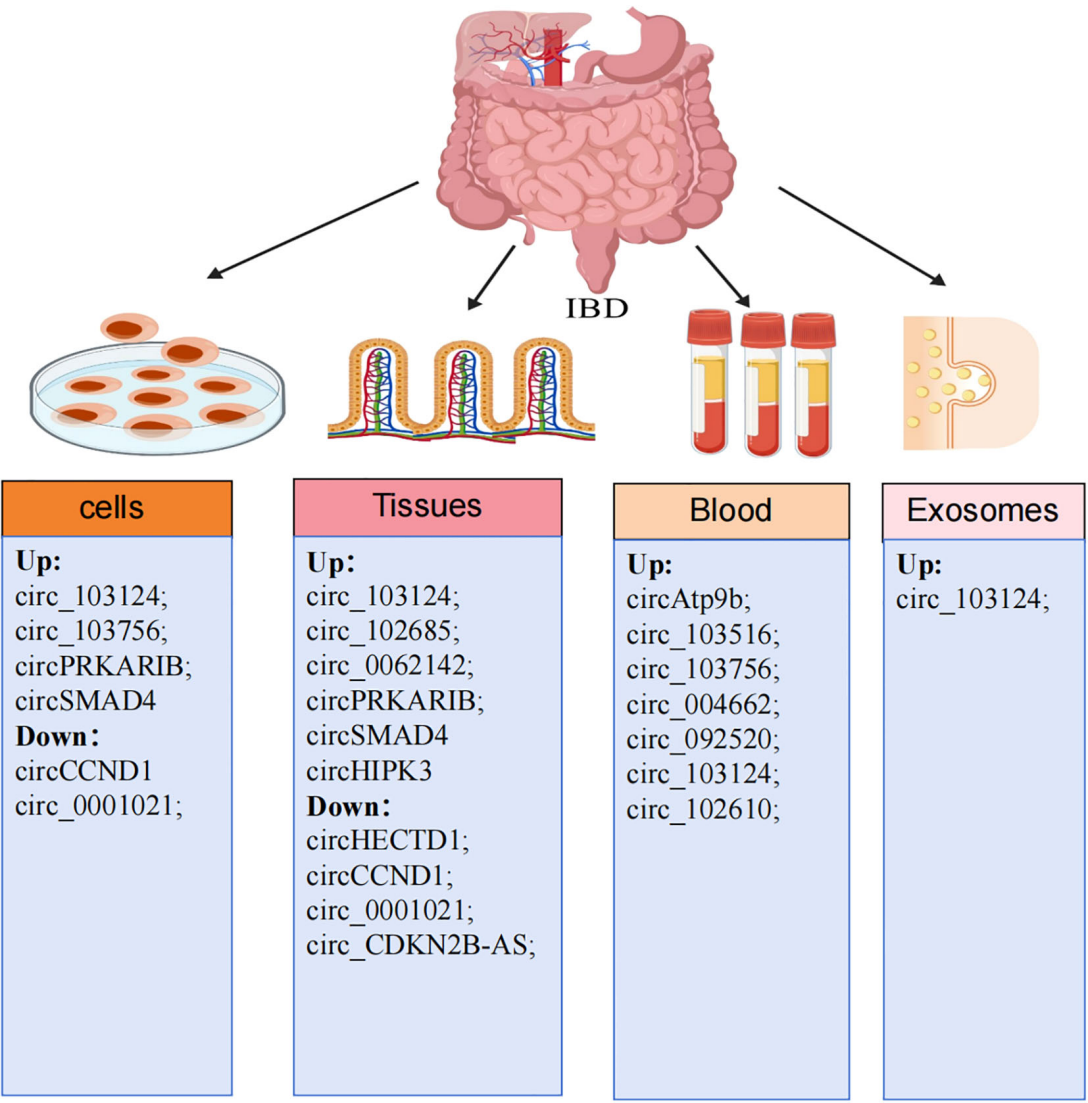
Aberrant expression of circRNAs in IBD patients. CircRNA shows aberrant expression in blood/serum, cells, colonic tissue and exosomes of IBD patients. Dysregulation of circRNA is highlighted in the figure; “up” indicates upregulation and “down” indicates downregulation.

Emerging evidence positions circRNAs as pivotal regulators in autoimmune disorders, particularly IBD. Moreover, circRNAs regulate various physiological and pathological mechanisms, and their differential expression during disease progression underscores their significance (25)(**Figure 1**). Elevated levels of circRNA_103516, circAtp9b, and circ_103756 in peripheral blood mononuclear cells (PBMCs) of IBD patients are associated with systemic inflammation (21, 26, 27). Exosomal circ_103124 is markedly upregulated in UC plasma, where it accelerates epithelial barrier disruption (28). Yin et al. found significant changes in four circRNAs(circ_092520, circ_102610,circ_004662,circ_103124)in PBMCs of CD patients, with circRNA_004662 in PBMCs distinguishes CD from UC, offering a non-invasive diagnostic tool (29). Importantly, recent studies highlight disease phase-dependent circRNA signature. Recent studies have further elucidated that circRNAs exhibit phase-specific expression dynamics during disease progression, with distinct profiles observed between active inflammatory phases and clinical remission.circ_103516 and circ_004662 are significantly elevated in IBD during active inflammation compared to remission (21, 22).CircHIPK3 is transiently upregulated in acute mucosal injury(e.g., cecal ligation and puncture models) to promote repair, but paradoxically downregulated in chronic IBD, correlating with impaired healing (30). Upregulation of circ_102685, circ_0062142, circSMAD4, circPRKARIB, and circ103124 in the intestinal mucosa tissues of CD patients and modulates key pathways (p53, TLR/NF-κB, IL-17, and JAK2/STAT3), perpetuating chronic inflammation (29, 31–34). Downregulation of circCCND1, circ_0001021, circHECTD1, and circ_CDKN2B-AS1 in colonic epithelial tissues of ulcerative colitis patients, while restoring their expression suppresses UC progression (35–38).

**Figure 2:**
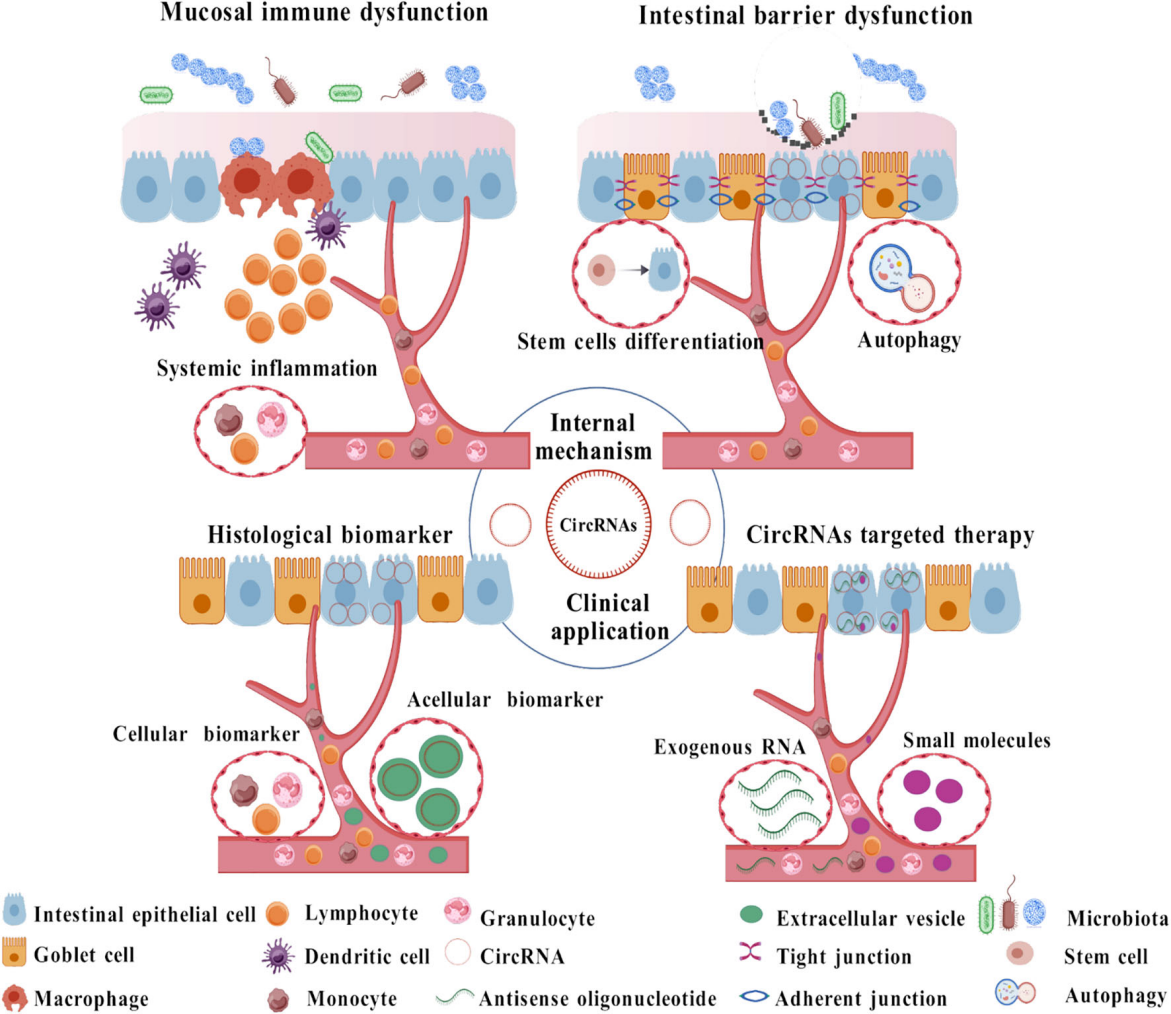
Mechanistic roles of circRNAs in IBD. Roles of circRNA in IBD are roughly classified into internal mechanisms and clinical applications (7). Internal mechanisms mainly focus on circRNAs regulating immune homeostasis and the intestinal barrier. clinical applications mainly include biomarkers and therapeutic predictors. circRNAs can also function as therapeutic targets of IBD with continued research and technique development. circRNA circular RNA, IBD inflammatory bowel disease.

CircRNA aberrant expression correlates with disease severity, inflammatory intensity, and phase-specific progression, exhibiting mechanistic roles in mucosal homeostasis and immune dysregulation (**Figure 2**). These findings contribute to a deeper understanding of the molecular mechanism of IBD and provide novel directions for diagnosis and treatment.

## 3 Mechanistic roles of circRNAs in IBD pathogenesis

The pathogenesis of inflammatory bowel disease (IBD) is not yet fully understood. Many studies suggest that IBD is a multifactorial disease caused by various factors, including genetic defects, environmental abnormalities, dysbiosis of the gut microbiota, and autophagy, which can disrupt intestinal homeostasis and trigger immune-mediated inflammation in the gut (39–43). CircRNAs influence biological processes through multiple mechanisms: by acting as “sponges” for miRNAs, interacting with RNA-binding proteins (RBPs) to modulate RNA stability, post-transcriptional gene expression regulation (44, 45). Therefore, analyzing the expression changes and functions of circRNA in IBD helps to uncover its molecular mechanisms in the disease progression, providing valuable insights into understanding IBD pathogenesis and developing new therapeutic strategies. Emerging evidence underscores the pivotal involvement of circRNAs in the pathogenesis and progression of IBD, as summarized in **Table 1**.

**Table 1:** Summary of dysregulated CircRNA expression in IBD.

Mechanism	CircRNA	MiRNA	Source	Up/Down	Function	Clinical value	Disease activity	Ref
miRNA sponges	Circ_103516	MiR-19b-1-5p	PBMC	Up	Promotes intestinal inflammation	Diagnosis	Positive	(21)
	Circ_103756	MiR-30	PBMC/Cells	Up	Promotes apoptosis and intestinal inflammation	Therapeutic target	Positive	(27)
	Circ_102685	MiR-146	Tissues	Up	Promotes apoptosis and intestinal inflammation	Diagnosis/Therapeutic	Positive	(31, 49, 50)
	CircSMAD4	MiR-135a-5p	Tissues/Cells	Up	Disruption of the intestinal epithelial barrier, pro-inflammatory, immune disorders	Therapeutic target	Positive	(33)
	CircCCND1	MiR-142-5p	Tissues/Cells	Down	Inhibits inflammation and apoptosis	Therapeutic target	Negatively	(35)
	CircSnx5	miR-544	Tissues/Cells	Down	Promotes DC activation and inflammatory phenotype	Therapeutic target	Negatively	(54)
	Circ_0520	MiR-709		Up		Diagnosis	Negatively	(51)
	Circ_2243							
	Circ_103124	MiR-650/MiR-1236-3P	PMBC/Cells/Tissues	Up	Promotes of macrophage M1 polarization, pro-inflammatory, pyroptosis inhibition of autophagy	Diagnosis Therapeutic target	Positive	(52, 53)
	Circ_SoD2	MiR-223/MiR-378g	Tissues/Cells	Up	Promotes intestinal inflammation and compromises the intestinal barrier	Therapeutic target	Positive	(55, 56)
	CircHIPK3	MiR-29b	Tissues/Cells	Down	Promotes the intestinal barrier	Therapeutic target		(30)
	Circ_0001021	MiR-224-5p	Tissues/Cells	Down	Promotes the intestinal barrier	Therapeutic target	Negatively	(22)
	Circ_0007919	MiR-138	Tissues/Cells	Down	Promotes the intestinal barrier	Diagnosis Therapeutic target	Negatively	(36)
	CircGMCL1	MiR-124-3p	Tissues	Down	Promotes autophagy, inhibits pyroptosis, inflammation	Therapeutic target	Negatively	(58)
	CircHECTD1	MiR-182-5p	Tissues	Down	Promotes autophagy, inhibits inflammation	Therapeutic target	Negatively	(37)
RNA-Binding Protein Interaction	CircPan3		Tissues		Promotes ISC self-renewal and differentiation	Therapeutic target		(61)
	CircBtnl1		Tissues		Inhibits ISC self-renewal and differentiation			(34)
	circPABPN1		Tissues	Up	Inhibits autophagy, Promotes inflammation	Therapeutic target	Positive	(65)
	CircPRKAR1B		Tissues/Cells	Up	Interact with SPTBN1,inhibits autophagy, promotes pyroptosis and inflammation	Therapeutic target	Positive	(66)
Epigenetic Modulation	CircPRKAR1B		Tissues/Cells	Up	(m6A) modification,inhibits autophagy, promotes pyroptosis and inflammation	Therapeutic target	Positive	(66)

PBMC, Peripheral blood mononuclear cells; SPTBN1, Spectrin beta non-erythrocytic 1.

### 3.1 CircRNAs as microRNA sponge

CircRNAs harbor multiple miRNA-binding sites, functioning as molecular “sponges” to sequester miRNAs, amplifying downstream gene expression. This regulatory axis termed the competing endogenous RNA (ceRNA) mechanism, is pivotal in modulating IBD pathogenesis. CircRNAs promote the progression of IBD by sequestering miRNAs to alleviate their suppression of target mRNAs and driving pathological processes through pro-inflammatory signaling pathways, intestinal epithelial dysfunction, and autophagy regulation.

#### 3.1.1 Pro-inflammatory signaling and intestinal immune homeostasis

CircRNAs serve as central regulators of inflammatory cytokine production and immune responses, critically influencing the pathogenesis of inflammatory diseases (46). For instance, the expression level of circ_103516 is elevated in active CD and UC patients, its expression positively correlates with TNF-α and IFN-γ levels while inversely associating with IL-10 (21). Mechanistically, it acts as a sponge for hsa-miRNA-19b-1-5p, participating in the inflammatory process of CD (47).In CD, circ_103765 acts as a ceRNA for the miR-30 family, exerting a pro-inflammatory role (27). The imbalance between Treg and Th17 cells is a key factor in the development of CD (48). circSMAD4 functions as a ceRNA by sequestering miR-135a-5p, activating JAK2/STAT3 signaling to promote Th1/Th17 differentiation. Therapeutic inhibition of circSMAD4 restores Treg/Th17 equilibrium, attenuating intestinal inflammation (33). Research conducted by Qiao has revealed that the expression of circ_102685 is increased in CD, and through influencing the circ_102685/miRNA-146/NF-κB signaling axis, it regulates T regulatory cells (Treg cells) and Dendritic Cells (DCs) to alleviate intestinal inflammation (31, 49, 50).In melatonin-treated colitis models,circ_0520 and circ_2243 impair bone marrow-derived dendritic cells(BMDCs) maturation by targeting YWHAZ/CCL19 through the PI3K/AKT pathway, limiting DC phagocytosis and migration and production of pro-inflammatory cytokines, thereby alleviating intestinal inflammation (51). A study by Yin et al. found that circ_103124 functions as a molecular sponge for miR-650 to activate AKT2/TLR4/NF-κB signaling pathways, driving M1 macrophage polarization. Its upregulation in active CD correlates with TLR4(Toll-like receptor 4)/MyD88(Myeloid differentiation factor 88)/NLRP3/GSDMD axis activation (Gasdermin D), perpetuating pyroptosis and inflammation (52, 53). CircSnx5 acts as a sponge for miR-544 to upregulate the suppressor of cytokine signaling 1 (SOCS1), suppressing the nuclear translocation of PU.1 and DC-mediated T-cell activation. This mechanism highlights its potential in treating IBD and autoimmune disorders (54).CircRNACCND1 sponges miR-142-5p, thereby alleviating its inhibitory effect on Nuclear Receptor Coactivator 3(NCOA3) and restoring NCOA3-mediated anti-inflammatory activity (35).

#### 3.1.2 Intestinal epithelial barrier dysfunction

CircRNAs play a key role in preserving the intestinal barrier, interacting with tight junction proteins to exert dual functions. For example, circ_SOD2 affects CLDN-8 or damages the intestinal barrier via the miR-378g/Snail1 pathway, promoting the progression of ulcerative colitis (55, 56).circSMAD4 functions as a ceRNA to sequester miR-135a-5p, leading to JAK2 overexpression and subsequent degradation of tight junction proteins (occludin, ZO-1). This mechanism drives epithelial barrier dysfunction in CD (33). Genome-wide analyses have revealed that circHIPK3 significantly downregulated in IBD and sepsis patients, circHIPK3 sponges miR-29b to derepress pro-repair effectors (Rac1, Cdc42, Cyclin B1), restoring the migratory and proliferative capacities of intestinal epithelial cells (30).Specific circRNAs, such as,circ_0001021 and circ_0007919,preserve barrier integrity by modulating Smad4,ZO-1,CLDN-2,CLDN-8,VIPR1, and EPC1 expression through miRNA-dependent mechanisms, with particular therapeutic relevance in UC (22, 36).This ceRNA mechanism plays a pivotal role in repairing mucosal damage in IBD.

#### 3.1.3 Autophagy modulation

Autophagy is a critical intracellular biological process that plays an essential role in cell health and stress response. It is crucial for maintaining intestinal homeostasis, regulating immunity, and defending against pathogens. Dysregulation of autophagy is associated with various diseases, particularly when it occurs in intestinal epithelial cells, impairs mucosal immunity, and exacerbates chronic inflammation (57).circGMCL1 functioning as a molecular sponge for miR-124-3p, derepressing its target ANXA7 to enhance autophagic. This mechanism suppresses NLRP3 inflammasome activation and pyroptosis, thereby preserving barrier integrity and attenuating inflammation in CD (58). circHECTD1 sequesters miR-182-5p to stabilize HuR, an RNA-binding protein that prolongs the half-life of ATG5 and ATG9 mRNAs in Caco-2 cells. Elevated ATG5/ATG9 levels rescue impaired autophagosome formation, mitigating oxidative stress and epithelial apoptosis in UC (37). Autophagy-related genes ATG5 and ATG9 play pivotal roles in UC pathogenesis. ATG5, a critical regulator of autophagy, not only maintains cellular homeostasis but also modulates immune responses and apoptosis. Its elevated expression in UC patients suggests its involvement in disease progression. Concurrently, ATG9 facilitates autophagosome membrane formation during autophagy, further underscoring the centrality of autophagic dysregulation in UC pathogenesis (59, 60). Collectively, these findings underscore the intricate interplay between autophagy and inflammatory regulation, highlighting their dynamic balance in maintaining tissue homeostasis and disease progression.

By sponging specific miRNAs, circRNAs establish intricate competing ceRNA regulatory networks, which orchestrate multiple pathological cascades in IBD progression: inflammatory amplification, immune homeostasis disruption, barrier dysfunction, and autophagy-inflammatory crosstalk. These synergistic mechanisms collectively perpetuate the sustained chronic inflammation and progressive tissue damage characteristic of IBD.

### 3.2 CircRNAs bind to RNA-binding protein (RBP): post-transcriptional control

CircRNAs can bind to RBPs and coordinate post-transcriptional networks in the pathogenesis of IBD. This mechanism may contribute to the development and progression of IBD by influencing intestinal epithelial cell proliferation, autophagy, and inflammatory signaling pathways.

#### 3.2.1 Intestinal stem cells-mediated intestinal mucosal repair

The intestinal epithelium serves as the primary barrier against luminal threats, with intestinal stem cells (ISCs) being indispensable for preserving mucosal homeostasis. Emerging evidence highlights the regulatory dominance of specific circRNAs in this biological process. For instance, circPan3 upregulation in Lgr5+ ISCs elevates IL-13Rα1 mRNA levels, enhancing their regenerative capacity and differentiation potential, thereby facilitating mucosal repair and epithelial barrier restoration. These findings underscore the pivotal role of the IL-13Rα1-mediated signaling axis in ISC-driven tissue regeneration. Conversely, genetic ablation of CircPan3 in Lgr5+ ISCs exacerbates intestinal inflammation in preclinical models, suggesting its protective function in mucosal immunity (61). In contrast, circBtnl1 demonstrates an inhibitory role in ISC dynamics. Mechanistically, it suppresses self-renewal by destabilizing Atf4 mRNA and downregulating Sox9 expression, a transcription factor essential for stem cell maintenance. This dual regulatory mechanism positions circBtnl1 as a negative modulator of ISC proliferation (34).

#### 3.2.2 Regulation of intestinal epithelial cell homeostasis

HuR (Human Antigen R), an RNA-binding protein, dynamically regulates gene expression through interactions with non-coding RNAs, including miRNAs and lncRNAs (62, 63). In the context of IBD, HuR binds to the 3’ untranslated region (3’UTR) of ATG16L1 mRNA, stabilizing its transcript and enhancing translational efficiency, thereby driving ATG16L1 protein synthesis. As a core component of autophagosome formation, ATG16L1 is essential for maintaining autophagic flux in intestinal epithelial cells, a critical process for epithelial homeostasis (64). Notably, emerging studies reveal HuR’s functional crosstalk with circRNAs. For example, circPABPN1 suppresses HuR-mediated translational activation of ATG16L1, impairing autophagy and triggering mucosal immune dysregulation. This HuR-circPABPN1-ATG16L1 axis is implicated in perpetuating intestinal inflammation and epithelial injury in IBD, highlighting its role as a pathogenic driver (65). Therapeutic targeting of this axis may restore autophagic activity and mitigate IBD progression. Furthermore, circPRKAR1B interacts with the RNA-binding protein SPTBN1 (spectrin beta non-erythrocytic 1), disrupting autophagy while activating pyroptosis, a pro-inflammatory cell death pathway. This dual mechanism exacerbates colonic inflammation in CD, underscoring the pathological relevance of circRNA-RBP complexes in IBD pathogenesis (66).

These insights underscore the centrality of circRNA-RBP interactions in IBD pathophysiology, bridging transcriptional regulation with epithelial-immune dysfunction. Their therapeutic exploitation holds transformative potential for halting disease progression.

### 3.3 circRNAs and epigenetic modulation

CircRNAs exhibit enhanced stability due to their covalently closed structures, which confer resistance to RNase R-mediated degradation. Notably, the metabolic dynamics of circRNAs are modulated through N6-methyladenosine (m6A)-dependent epigenetic regulatory mechanisms, a dynamic RNA modification that governs diverse biological processes, including RNA decay, circRNA biogenesis, splicing, and translational regulation. Notably, m6A modifications are prevalent across circRNAs and play a critical role in IBD pathogenesis. METTL3, the core catalytic subunit of the m6A methyltransferase complex, acts as a master “writer” of this modification. Elevated METTL3 expression has been observed in colonic tissues of CD patients compared to healthy controls, suggesting its involvement in IBD-related epigenetic dysregulation (67–70). For instance, circPRKAR1B undergoes METTL3-orchestrated m6A modification, which destabilizes its structure and accelerates degradation. This m6A-dependent regulation suppresses autophagic flux while activating the NLRP3 inflammasome-pyroptosis, thereby amplifying mucosal inflammation and epithelial damage in CD (66).

## 4 CircRNAs and intestinal fibrosis

Intestinal fibrosis represents a frequent complication in CD, where epithelial-mesenchymal transition (EMT) facilitates fibroblast recruitment within inflammatory foci. These fibroblasts constitute a principal reservoir of mesenchymal stromal cells(MSC) in the colonic mucosa (71).

Emerging evidence implicates circRNAs as pivotal regulators of fibrogenesis through EMT modulation. For instance, hsa_circ_0062142 and hsa_circ_0001666 critically influence CD pathogenesis by regulating EMT-mediated transcriptional reprogramming, thereby accelerating disease progression (32). Genetic depletion of circFOXP1 markedly impairs the fibrogenic differentiation potential of mesenchymal stem cells, as demonstrated in both experimental models and clinical specimens (72). Juan et al. revealed that hsa_circRNA_102610 functions as a competitive endogenous RNA by sequestering hsa-miR-130a-3p, which amplifies TGF-β1 signaling to potentiate EMT and extracellular matrix remodeling, ultimately driving intestinal fibrosis in CD (73).

Emerging evidence delineates a central role for circRNAs in driving intestinal fibrogenesis through coordinated regulation of EMT, TG-β1signaling hyperactivation, and MSC differentiation.

## 5 CircRNA and colitis-associated cancer(CAC)

Chronic inflammatory microenvironments serve as pivotal drivers of tumorigenesis, particularly in IBD, where persistent mucosal inflammation significantly elevates the risk of CRC (74). This review systematically integrates, for the first time, the molecular regulatory network by which circRNAs drive CAC pathogenesis. Comparative transcriptomic analyses reveal distinct circRNA expression profiles between UC, CAC, and normal colonic tissues, with specific circRNAs emerging as modulators of malignant transformation (22, 23). A study compared the expression of circRNAs in normal colon tissue versus the CAC model in mice. Key findings include: mmu_circ_001801, mmu_circ_002987, and mmu_circ_001155 were upregulated, while mmu_circ_000287, mmu_circ_003037, and mmu_circ_001226 were downregulated. Among these, mmu_circ_001801 and mmu_circ_003037 exhibit high sequence conservation with human orthologs, while mmu_circ_001226 and mmu_circ_000287 potentially regulate CAC via miRNA-mRNA networks (75). CircHIPK2 acts as a central node in Hippo pathway dysregulation. Through the circHIPK2-EIF4A3 axis, it enhances TAZ translation, promoting colonic epithelial hyperproliferation in both colitis and CRC (76). Three PRKAR2A-derived circRNAs (e.g., circPRKAR2A-1/2), sharing high homology with murine mmu_circ_0001845, are markedly upregulated in CAC tissues. These circRNAs accelerate the colitis-to-CAC transition and hold promise as diagnostic biomarkers and therapeutic targets for inflammation-driven malignancies (77) (**Table 2**).

**Table 2:** CircRNA and colitis-associated cancer.

Disease	circRNA	miRNA	Source	Up/Down	mechanism	Ref
AOM/DSS-induced colon carcinoma	mmu_circ_001801	Not report	Mice colonic tissuess	Up	involved in CAC	(75)
	mmu_circ_002987			Up		
	mmu_circ_001155			Up		
	mmu_circ_000287			Down		
	mmu_circ_003037			Down		
	mmu_circ_001226			Down		
	circHIPK2			Up		(76)
	circPRKAR2A-1/2			Up		(77)
	mmu_circ_0001845			Up		

**Figure 3:**
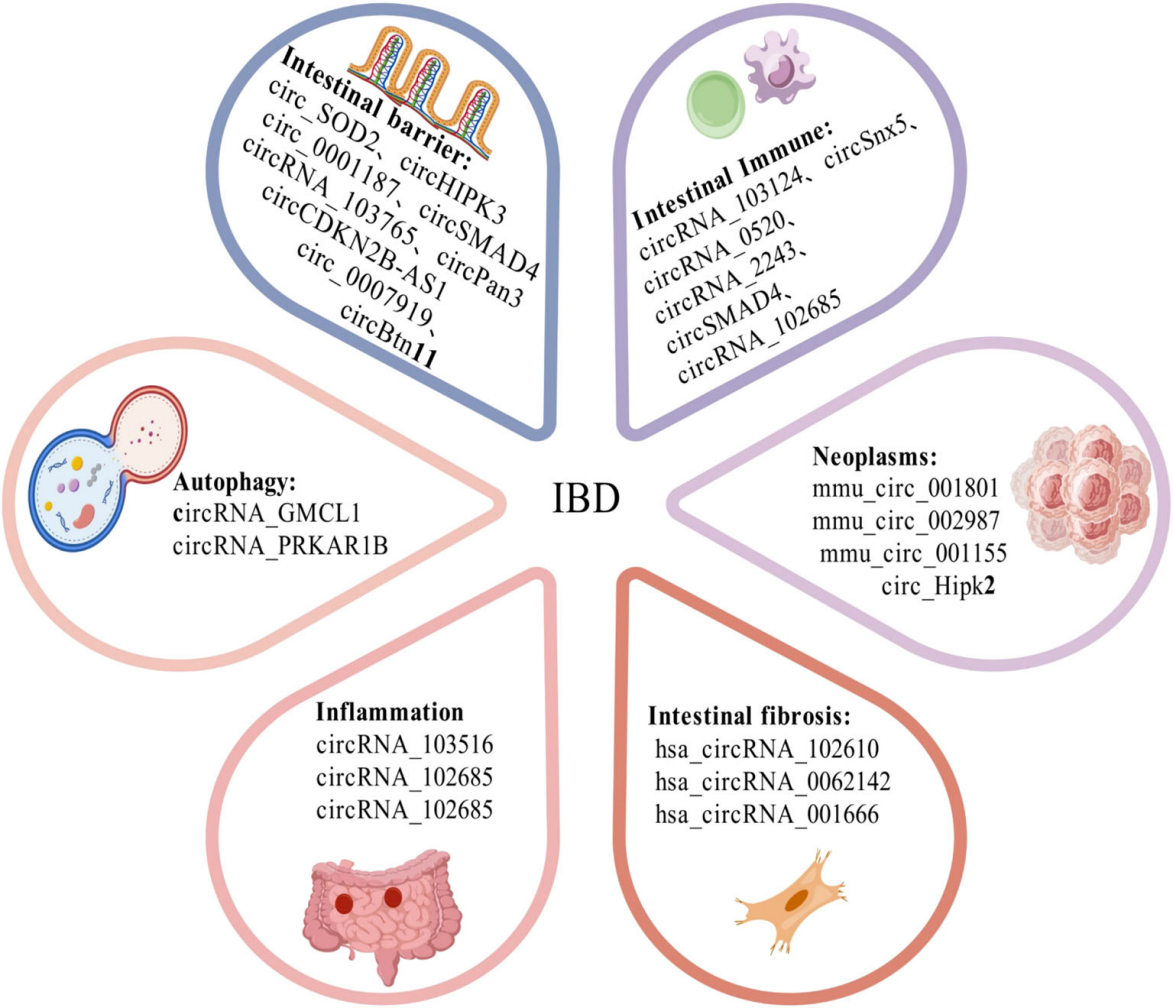
The pathogenic mechanisms of circRNAs in IBD. CircRNA has been found to be associated with the regulation of intestinal barrier function, autophagy, immune cell activity, and the modulation of inflammatory responses, as well as the progression of intestinal fibrosis and colitis-associated cancer (CAC). CircRNA, circular RNA; IBD, inflammatory bowel disease; CAC, colitis-associated cancer.

AOM azoxymethane,DSS dextran sulfate sodium,CAC colitis-associated cancer;

Emerging evidence underscores the central role of circRNAs in IBD pathogenesis, involving epithelial barrier integrity, stem cell regulation, immune cell activity, and regulation of autophagy and inflammatory response. Furthermore, circRNAs are also associated with the progression of intestinal fibrosis and Colitis-associated Cancer, offering new perspectives for the diagnosis and treatment of IBD (**Figure 3** **).**

## 6 CircRNAs as potential biomarkers for diagnosis and prognosis of IBD

While endoscopy with biopsy remains the primary and most effective diagnostic modality for IBD due to the absence of a gold standard. Epigenetic biomarkers, particularly in tissue, biofluids (serum, plasma), and exosomes are revolutionizing disease management through non-invasive, cost-efficient monitoring of diagnosis, prognosis, and treatment response.

**Figure 4:**
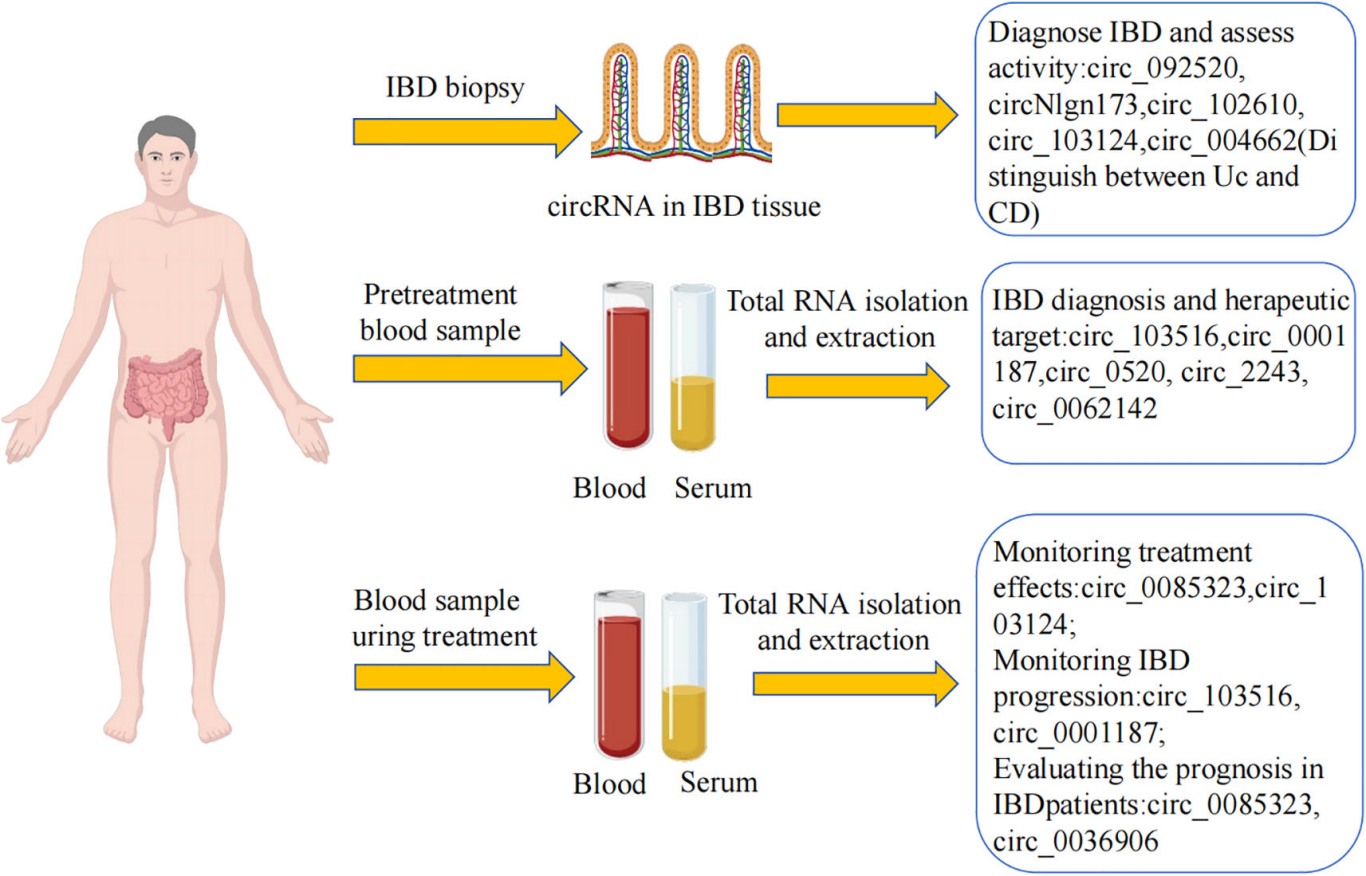
CircRNAs can be used as potential biomarkers for the diagnosis and treatment of IBD. CircRNAs can be used as indicators to differentiate between UC and CD. CircRNA expression levels in serum can serve as an ideal biomarker for the diagnosis of IBD and provide clinicians with a basis for developing appropriate therapeutic regimens. CircRNA levels in blood and serum are also important clinical markers for IBD monitoring, treatment and prognosis.

CircRNAs emerge as superior biomarkers owing to their structural stability, evolutionary conservation across species, tissue-/phase-specific expression patterns, high abundance in bodily fluids, and can be detectable via qRT-PCR (**Figure 4**). Studies have pointed out that specific circRNAs may serve as biomarkers for IBD. For example, in PBMCs of CD patients, circ_004662, circ_092520, circ_102610, and circ_103124 are significantly elevated in CD patients. Notably, circ_004662 achieves an AUC of 0.85 for distinguishing CD from UC (29). circ_103516 levels in PBMCs correlate positively with CD activity indices (CDAI) (21). Drives UC progression via the miR-1236-3p/MYD88 axis, serving as a dual potential diagnostic and therapeutic target.

Circ_0001187, through interaction with the miRNA-1236-3p/MYD88 pathway, promotes the development of UC and is a potential diagnostic and therapeutic biomarker (28).circNlgn173 is upregulated in active UC mucosa (78). Additionally, circ_0520, circ_2243, circ_0062142, and circ_0001666 have also been identified for their diagnostic potential in IBD (32, 51).

CircRNAs can also be used for prognosis assessment. For example, circ_0085323 and circ_0036906 predict clinical outcomes of UC (79).

## 7 Therapeutic targeting of circRNAs in inflammatory bowel disease: emerging strategies

CircRNAs are increasingly recognized as dynamic regulators of IBD pathogenesis, with their dysregulation influencing transcriptional networks and disease progression. This section delineates cutting-edge approaches for circRNA modulation, siRNA or shRNA silencing, overexpression, and innovative delivery systems.

### 7.1 RNA interference-mediated circRNA knockdown

RNA interference technology achieves the knockdown of circRNAs through small interfering RNA (siRNA) or short hairpin RNA (shRNA). For example, Silencing circ_0085323 via siRNA suppresses TNF-α-induced epithelial damage by derepressing miR-495-3p/TRAF3 signaling, demonstrating therapeutic efficacy for UC (80). ShRNA-mediated inhibition of hsa_circ_103124 enhances autophagy, reduces NLRP3 inflammasome activation, and mitigates intestinal inflammation in CD through miR-650 sequestration, making it a potential therapeutic target (52, 53).

### 7.2 Viral vector-mediated circRNA overexpression

CircRNA expression vectors exploit endogenous back-splicing mechanisms driven by complementary intronic sequences and RNA-binding proteins (RBPs). Lentivirus and adenovirus vectors are commonly used for *in vivo* overexpression of circRNAs (81). For example, in UC, restoration of circRNA CCND1 via lentiviral vectors inhibits LPS-induced apoptosis in Caco-2 cells and mitigates inflammatory responses, preserving epithelial integrity (35). These findings provide experimental evidence for the development of circRNA-based targeted therapies for UC.

### 7.3 Biologics targeting circRNA-inflammatory crosstalk

Biological agents, such as infliximab, a chimeric monoclonal antibody of human and mouse origin, are TNF-α inhibitors. They are widely used in treating autoimmune diseases like IBD, ankylosing spondylitis, and psoriatic arthritis, and are known for their high efficacy, safety, and low incidence of side effects (82). Infliximab can reverses circ_103765 upregulation in CD mucosa, suggesting synergy between biologics and circRNA modulation (27). However, Clinical efficacy in circRNA-high IBD requires validation through multicenter trials.

### 7.4 Poly (lactic-co-glycolic acid)-microspheres (PLGA MSs) delivery systems

PLGA MSs enhance low-dose drug bioavailability and enable colon-targeted circRNA delivery. PLGA MSs loaded with circGMCL1 mimics restore autophagy and inhibit pyroptosis via the miR-124-3p/ANXA7 axis, alleviating barrier dysfunction in CD (58). In animal model experiments, PLGA MSs-si-circSMAD4 treatment reduces colitis severity, evidenced by longer colon length and lower histologic inflammation scores, compared to the untreated control group (33).

### 7.5 Nanocarrier/exosome platforms

Research reports indicate that circRNA vaccines provide strong protection against SARS-CoV-2 in mice and rhesus monkeys (83). CircRNAs can be encapsulated and delivered via nanocarriers and exosomes, which may enhance the efficacy of therapeutic drugs (84, 85). However, whether circRNAs can be clinically translated for the treatment of IBD in the medical field requires further investigation.

## 8 Discussion and future perspectives

Recent advances have elucidated the multifaceted roles of circRNAs in IBD, offering novel insights into disease pathogenesis and unveiling potential therapeutic avenues. CircRNAs exhibit dysregulated expression in intestinal tissues, PBMCs, and exosomes of patients with UC and CD (28–30). These molecules orchestrate IBD progression through diverse mechanisms: acting as miRNA sponges (e.g., circ_103516/miR-19b-1-5p axis), recruiting RNA-binding proteins (RBPs;e.g., HuR/circPABPN1/ATG16L1axis), and epigenetic modulation(e.g.,circPRKAR1B) (47, 65, 66). Such interactions drive pathological cascades, including driving inflammation, intestinal barrier dysfunction, intestinal immune homeostasis, autophagy, EMT, intestinal fibrosis, and Colitis-associated cancer. Notably, phase-specific circRNA signatures (e.g., elevated circ_103516 in active IBD vs. remission) highlight their utility as non-invasive biomarkers for disease monitoring (21). Furthermore, circRNA-based therapeutic strategies such as PLGA-microsphere-mediated delivery and RNA interference-based silencing can attenuate colitis severity in murine models.

The mechanistic roles of circRNAs in IBD is gaining significant attention. Although its mechanisms are not yet fully understood, its key role in various diseases and potential as biomarkers have already become evident. Despite progress, critical gaps hinder clinical translation.

Current RNA sequencing (RNA-seq) struggles to differentiate circRNAs from linear isoforms due to sequence overlap, while ribosome profiling fails to conclusively confirm their translational potential (86). Bioinformatics tools(e.g., CIRI, CIRCexplorer) exhibit reduced sensitivity in low-abundance biofluids(e.g., fecal or serum samples) (87).

While circRNAs (e.g., circAtp9b, circ_103516, circ_103124) correlate with IBD activity, their expression in non-IBD gastrointestinal disorders (e.g., infectious or allergic colitis) remains unvalidated. Large-scale multicenter studies are imperative to establish diagnostic thresholds and address confounding variables (e.g., microbiome composition, and medication effects). Additionally, circRNA profiles in urine, saliva, and stool, which are key non-invasive sources, are still lacking comprehensive characterization, thus limiting their diagnostic utility.

Systemic delivery of circRNA mimics or inhibitors faces challenges, including nuclease degradation, off-target effects, nonspecific tissue or cell type targeting, Mis-spliced products, and so on (83). Innovations in nanocarriers (e.g., exosome-based delivery) or engineered circRNA may enhance local bioavailability (85, 88). Immunogenicity risks of viral vectors (e.g., adenovirus) require careful evaluation. Non-viral delivery systems, such as lipid nanoparticles represent promising alternatives (84). Despite challenges in the use of circRNA drug delivery systems for the treatment of IBD, their potential in precision medicine and disease management should not be overlooked.

Breakthroughs in high-throughput sequencing and bioinformatic analytics have revolutionized our understanding of circRNAs, unveiling their previously unrecognized functionalities and establishing them as pivotal targets in contemporary biomedical research. Combining circRNA profiling with epigenomic, proteomic, and metabolomic datasets will unravel IBD complex regulatory networks. Single-cell and spatial transcriptomics can resolve cell type-specific circRNA functions within inflammatory niches. CRISPR-based tools (e.g., Cas9, Cas13d) enable targeted circRNA knockdown *in vivo*, offering a platform to validate therapeutic targets (89, 90). However, the long-term safety evaluation of circRNA-based therapeutics must prioritize the following risks:(1) Preventing unintended genomic integration of exogenous circRNAs, which may induce mutagenesis or carcinogenesis. (2) Mitigating excessive immune activation triggered by immunostimulatory circRNA motifs. (3) Minimizing prolonged retention in clearance organs (e.g., liver, kidneys) to avoid dose-dependent toxicity and organ damage.

## 9 Conclusions

This review systematically delineates the expression profiles and mechanistic roles of circRNAs in IBD pathogenesis. It underscores their dual utility as non-invasive diagnostic and prognostic biomarkers and therapeutic targets via modulation of circRNA-driven regulatory networks. Future breakthroughs in IBD therapeutics will rely on concerted interdisciplinary efforts to orchestrate circRNA-targeted strategies to transform molecular insights into clinically actionable solutions, thereby advancing personalized medicine frameworks for IBD.

## References

[1] Ananthakrishnan AN Kaplan GG Ng SC. Changing global epidemiology of inflammatory bowel diseases: sustaining health care delivery into the 21st century. Clin gastroenterol hepatol. (2020) 18:1252–60. doi: 10.1016/j.cgh.2020.01.028 32007542

[2] Desai D Dhoble P. Rapidly changing epidemiology of inflammatory bowel disease: Time to gear up for the challenge before it is too late. Indian J gastroenterol. (2024) 43:15–7. doi: 10.1007/s12664-023-01453-6 37773577

[3] Fumery M Yzet C Chatelain D Yzet T Brazier F LeMouel JP. Colonic strictures in inflammatory bowel disease: epidemiology, complications, and management. J Crohn’s colitis. (2021) 15:1766–73. doi: 10.1093/ecco-jcc/jjab068 33844013

[4] Yao RW Wang Y Chen LL. Cellular functions of long noncoding RNAs. Nat Cell Biol. (2019) 21:542–51. doi: 10.1038/s41556-019-0311-8 31048766

[5] Pourmehran Y Sadri F Hosseini SF Mohammadi Y Rezaei Z. Exploring the influence of non-coding RNAs on NF-κB signaling pathway regulation in ulcerative colitis. Biomed pharmacother = Biomed pharmacother. (2024) 179:117390. doi: 10.1016/j.biopha.2024.117390 39243424

[6] Hu Y Lu Y Fang Y Zhang Q Zheng Z Zheng X. Role of long non-coding RNA in inflammatory bowel disease. Front Immunol. (2024) 15:1406538. doi: 10.3389/fimmu.2024.1406538 38895124 PMC11183289

[7] Lin L Zhou G Chen P Wang Y Han J Chen M. Which long noncoding RNAs and circular RNAs contribute to inflammatory bowel disease? Cell Death Dis. (2020) 11:456. doi: 10.1038/s41419-020-2657-z 32541691 PMC7295799

[8] Liu CX Chen LL. Circular RNAs: Characterization, cellular roles, and applications. Cell. (2022) 185:2016–34. doi: 10.1016/j.cell.2022.04.021 35584701

[9] Aufiero S Reckman YJ Pinto YM Creemers EE. Circular RNAs open a new chapter in cardiovascular biology. Nat Rev Cardiol. (2019) 16:503–14. doi: 10.1038/s41569-019-0185-2 30952956

[10] Vromman M Vandesompele J Volders PJ. Closing the circle: current state and perspectives of circular RNA databases. Briefings Bioinf. (2021) 22:288–97. doi: 10.1093/bib/bbz175 PMC7820840 31998941

[11] Li J Wang X. Functional roles of conserved lncRNAs and circRNAs in eukaryotes. Non-coding RNA Res. (2024) 9:1271–9. doi: 10.1016/j.ncrna.2024.06.014 PMC11260338 39036601

[12] Ma Y Wang T Zhang X Wang P Long F. The role of circular RNAs in regulating resistance to cancer immunotherapy: mechanisms and implications. Cell Death dis. (2024) 15:312. doi: 10.1038/s41419-024-06698-3 38697964 PMC11066075

[13] García-Rodríguez JL Korsgaard U Ahmadov U Jarlstad Olesen MT Dietrich KG Hansen EB. Spatial profiling of circular RNAs in cancer reveals high expression in muscle and stromal cells. Cancer Res. (2023) 83:3340–53. doi: 10.1158/0008-5472.CAN-23-0748 PMC10570686 37477923

[14] Mei X Chen SY. Circular RNAs in cardiovascular diseases. Pharmacol Ther. (2022) 232:107991. doi: 10.1016/j.pharmthera.2021.107991 34592203 PMC8930437

[15] Zhao RJ Zhang WY Fan XX. Circular RNAs: Potential biomarkers and therapeutic targets for autoimmune diseases. Heliyon. (2024) 10:e23694. doi: 10.1016/j.heliyon.2023.e23694 38205329 PMC10776946

[16] Xiao F He Z Wang S Li J Fan X Yan T. Regulatory mechanism of circular RNAs in neurodegenerative diseases. CNS Neurosci Ther. (2024) 30:e14499. doi: 10.1111/cns.14499 37864389 PMC11017410

[17] Kim E Kim YK Lee SV. Emerging functions of circular RNA in aging. Trends gene: TIG. (2021) 37:819–29. doi: 10.1016/j.tig.2021.04.014 34016449

[18] Jagtap U Anderson ES Slack FJ. The emerging value of circular noncoding RNA research in cancer diagnosis and treatment. Cancer Res. (2023) 83:809–13. doi: 10.1158/0008-5472.CAN-22-3014 PMC10020866 36919419

[19] Liu Q Li S. Exosomal circRNAs: Novel biomarkers and therapeutic targets for urinary tumors. Cancer lett. (2024) 588:216759. doi: 10.1016/j.canlet.2024.216759 38417667

[20] Lin Z Ji Y Zhou J Li G Wu Y Liu W. Exosomal circRNAs in cancer: Implications for therapy resistance and biomarkers. Cancer lett. (2023) 566:216245. doi: 10.1016/j.canlet.2023.216245 37247772

[21] Ye YL Yin J Hu T Zhang LP Wu LY Pang Z. Increased circulating circular RNA_103516 is a novel biomarker for inflammatory bowel disease in adult patients. World J gastroenterol. (2019) 25:6273–88. doi: 10.3748/wjg.v25.i41.6273 PMC6848015 31749597

[22] Wang T Chen N Ren W Liu F Gao F Ye L. Integrated analysis of circRNAs and mRNAs expression profile revealed the involvement of hsa_circ_0007919 in the pathogenesis of ulcerative colitis. J gastroenterol. (2019) 54:804–18. doi: 10.1007/s00535-019-01585-7 31037450

[23] Qiu C Chen Y Xia H Duan J Zhang L Zhang Y. Hsa_circ_0004662 accelerates the progression of ulcerative colitis via the microRNA-532/HMGB3 signalling axis. J Cell Mol Med. (2025) 29(6):e70430. doi: 10.1111/jcmm.70430 40099942 PMC11916553

[24] Lun J Guo J Yu M Zhang H Fang J. Circular RNAs in inflammatory bowel disease. Front Immunol. (2023) 14:1307985. doi: 10.3389/fimmu.2023.1307985 38187401 PMC10771839

[25] Yi Q Feng J Lan W Shi H Sun W Sun W. CircRNA and lncRNA-encoded peptide in diseases, an update review. Mol cancer. (2024) 23:214. doi: 10.1186/s12943-024-02131-7 39343883 PMC11441268

[26] Li F Fu J Fan L Lu S Zhang H Wang X. Overexpression of circAtp9b in ulcerative colitis is induced by lipopolysaccharides and upregulates PTEN to promote the apoptosis of colonic epithelial cells. Exp Ther med. (2021) 22:1404. doi: 10.3892/etm.2021.10840 34675997 PMC8524737

[27] Ye Y Zhang L Hu T Yin J Xu L Pang Z. CircRNA_103765 acts as a proinflammatory factor via sponging miR-30 family in Crohn’s disease. Sci reports. (2021) 11:565. doi: 10.1038/s41598-020-80663-w PMC7804428 33436852

[28] Ouyang W Wu M Wu A Xiao H. Circular RNA_0001187 participates in the regulation of ulcerative colitis development via upregulating myeloid differentiation factor 88. Bioengineered. (2022) 13:12863–75. doi: 10.1080/21655979.2022.2077572 PMC9275921 35609334

[29] Yin J Hu T Xu L Li P Li M Ye Y. Circular RNA expression profile in peripheral blood mononuclear cells from Crohn disease patients. Medicine. (2019) 98:e16072. doi: 10.1097/MD.0000000000016072 31261517 PMC6617429

[30] Xiao L Ma XX Luo J Chung HK Kwon MS Yu TX. Circular RNA circHIPK3 promotes homeostasis of the intestinal epithelium by reducing microRNA 29b function. Gastroenterology. (2021) 161:1303–17.e3. doi: 10.1053/j.gastro.2021.05.060 34116030 PMC8463477

[31] Qiao YQ Cai CW Shen J Zheng Q Ran ZH. Circular RNA expression alterations in colon tissues of Crohn’s disease patients. Mol Med reports. (2019) 19:4500–6. doi: 10.3892/mmr.2019.10070 30896837

[32] Hu YA Zhu Y Liu G Yao X Yan X Yang Y. Expression profiles of circular RNAs in colon biopsies from Crohn’s disease patients by microarray analysis. J Clin Lab anal. (2021) 35:e23788. doi: 10.1002/jcla.23788 33955043 PMC8183921

[33] Zhao J Lin Z Ying P Zhao Z Yang H Qian J. circSMAD4 promotes experimental colitis and impairs intestinal barrier functions by targeting janus kinase 2 through sponging miR-135a-5p. J Crohn’s colitis. (2023) 17:593–613. doi: 10.1093/ecco-jcc/jjac154 36239525

[34] Guo H Zhang J Jiang Z Zhu X Yang J Mu R. Noncoding RNA circBtnl1 suppresses self-renewal of intestinal stem cells via disruption of Atf4 mRNA stability. EMBO J. (2023) 42:e112039. doi: 10.15252/embj.2022112039 36715460 PMC10015366

[35] Xiang P Ge T Zhou J Zhang Y. Protective role of circRNA CCND1 in ulcerative colitis via miR-142-5p/NCOA3 axis. BMC gastroenterol. (2023) 23:18. doi: 10.1186/s12876-023-02641-6 36658474 PMC9850594

[36] Li B Li Y Li L Yu Y Gu X Liu C. Hsa_circ_0001021 regulates intestinal epithelial barrier function via sponging miR-224-5p in ulcerative colitis. Epigenomics. (2021) 13:1385–401. doi: 10.2217/epi-2021-0230 34528447

[37] Xu Y Tian Y Li F Wang Y Yang J Gong H. Circular RNA HECTD1 mitigates ulcerative colitis by promoting enterocyte autophagy via miR-182-5p/huR axis. Inflamm bowel dis. (2022) 28:273–88. doi: 10.1093/ibd/izab188 34427642

[38] Rankin CR Lokhandwala ZA Huang R Pekow J Pothoulakis C Padua D. Linear and circular CDKN2B-AS1 expression is associated with Inflammatory Bowel Disease and participates in intestinal barrier formation. Life Sci. (2019) 231:116571. doi: 10.1016/j.lfs.2019.116571 31207308 PMC6897550

[39] Ananthakrishnan AN Bernstein CN Iliopoulos D Macpherson A Neurath MF Ali RAR. Environmental triggers in IBD: a review of progress and evidence. Nat Rev Gastroenterol hepatol. (2018) 15:39–49. doi: 10.1038/nrgastro.2017.136 29018271

[40] Glassner KL Abraham BP Quigley EMM. The microbiome and inflammatory bowel disease. J Allergy Clin Immunol. (2020) 145:16–27. doi: 10.1016/j.jaci.2019.11.003 31910984

[41] Geremia A Biancheri P Allan P Corazza GR Di Sabatino A. Innate and adaptive immunity in inflammatory bowel disease. Autoimmun Rev. (2014) 13:3–10. doi: 10.1016/j.autrev.2013.06.004 23774107

[42] Larabi A Barnich N Nguyen HTT. New insights into the interplay between autophagy, gut microbiota and inflammatory responses in IBD. Autophagy. (2020) 16:38–51. doi: 10.1080/15548627.2019.1635384 31286804 PMC6984609

[43] Holmberg FE Seidelin JB Yin X Mead BE Tong Z Li Y. Culturing human intestinal stem cells for regenerative applications in the treatment of inflammatory bowel disease. EMBO Mol med. (2017) 9:558–70. doi: 10.15252/emmm.201607260 PMC5412884 28283650

[44] Misir S Wu N Yang BB. Specific expression and functions of circular RNAs. Cell Death different. (2022) 29:481–91. doi: 10.1038/s41418-022-00948-7 PMC8901656 35169296

[45] Qu S Yang X Li X Wang J Gao Y Shang R. Circular RNA: A new star of noncoding RNAs. Cancer lett. (2015) 365:141–8. doi: 10.1016/j.canlet.2015.06.003 26052092

[46] Ashrafizadeh M Zarrabi A Mostafavi E Aref AR Sethi G Wang L. Non-coding RNA-based regulation of inflammation. Semin Immunol. (2022) 59:101606. doi: 10.1016/j.smim.2022.101606 35691882

[47] Cheng X Zhang X Su J Zhang Y Zhou W Zhou J. miR-19b downregulates intestinal SOCS3 to reduce intestinal inflammation in Crohn’s disease. Sci reports. (2015) 5:10397. doi: 10.1038/srep10397 PMC4441154 25997679

[48] Zhao J Lu Q Liu Y Shi Z Hu L Zeng Z. Th17 cells in inflammatory bowel disease: cytokines, plasticity, and therapies. J Immunol Res. (2021) 2021:8816041. doi: 10.1155/2021/8816041 33553436 PMC7846404

[49] Nata T Fujiya M Ueno N Moriichi K Konishi H Tanabe H. MicroRNA-146b improves intestinal injury in mouse colitis by activating nuclear factor-κB and improving epithelial barrier function. J Gene Med. (2013) 15:249–60. doi: 10.1002/jgm.v15.6-7 23813877

[50] Xu XM Zhang HJ. miRNAs as new molecular insights into inflammatory bowel disease: Crucial regulators in autoimmunity and inflammation. World J gastroenterol. (2016) 22:2206–18. doi: 10.3748/wjg.v22.i7.2206 PMC4734997 26900285

[51] Feng S Xu Z Zhang Z Mo Y Deng Y Li L. RNA-Seq approach to investigate the effects of melatonin on bone marrow-derived dendritic cells from dextran sodium sulfate-induced colitis mice. Toxicology. (2022) 481:153354. doi: 10.1016/j.tox.2022.153354 36265525

[52] Yin J Hu T Xu L Zhang L Zhu J Ye Y. Hsa_circRNA_103124 upregulation in Crohn’s disease promoted macrophage M1 polarization to maintain an inflammatory microenvironment via activation of the AKT2 and TLR4/NF-κB pathways. Int immunopharmacol. (2023) 123:110763. doi: 10.1016/j.intimp.2023.110763 37567009

[53] Yin J Hu T Xu LJ Zhang LP Ye YL Pang Z. The mechanism by which hsa_circRNA_103124 highly expressed in peripheral blood of patients with active Crohn’s disease regulates macrophage differentiation, pyroptosis and inflammation. Zhonghua yi xue za zhi. (2023) 103:3478–86. doi: 10.3760/cma.j.cn112137-20231007-00646 37981775

[54] Chen Q Mang G Wu J Sun P Li T Zhang H. Circular RNA circSnx5 Controls Immunogenicity of Dendritic Cells through the miR-544/SOCS1 Axis and PU.1 Activity Regulation. Mol Ther. (2020) 28:2503–18. doi: 10.1016/j.ymthe.2020.07.001 PMC7646215 32681834

[55] Wang TT Han Y Gao FF Ye L Zhang YJ. Effects of circular RNA circ-SOD2 on intestinal epithelial barrier and ulcerative colitis. Beijing da xue xue bao Yi xue ban = J Peking Univ Health Sci. (2019) 51:805–12. doi: 10.19723/j.issn.1671-167X.2019.05.003 PMC7433506 31624381

[56] Ye G Zhang J Peng J Zhou Z Wang W Yao S. CircSOD2: Disruption of intestinal mucosal barrier function in ulcerative colitis by regulating the miR-378g/Snail1 axis. J gastroenterol hepatol. (2024) 39:1299–309. doi: 10.1111/jgh.16550 38646884

[57] Levine B Mizushima N Virgin HW. Autophagy in immunity and inflammation. Nature. (2011) 469:323–35. doi: 10.1038/nature09782 PMC3131688 21248839

[58] Zhao J Sun Y Yang H Qian J Zhou Y Gong Y. PLGA-microspheres-carried circGMCL1 protects against Crohn’s colitis through alleviating NLRP3 inflammasome-induced pyroptosis by promoting autophagy. Cell Death dis. (2022) 13:782. doi: 10.1038/s41419-022-05226-5 36088391 PMC9464224

[59] Ardali R Kazemipour N Nazifi S Bagheri Lankarani K Razeghian Jahromi I Sepehrimanesh M. Pathophysiological role of Atg5 in human ulcerative colitis. Intest Res. (2020) 18:421–9. doi: 10.5217/ir.2019.00120 PMC7609390 32380583

[60] Noda NN. Structural view on autophagosome formation. FEBS lett. (2024) 598:84–106. doi: 10.1002/1873-3468.14742 37758522

[61] Zhu P Zhu X Wu J He L Lu T Wang Y. IL-13 secreted by ILC2s promotes the self-renewal of intestinal stem cells through circular RNA circPan3. Nat Immunol. (2019) 20:183–94. doi: 10.1038/s41590-018-0297-6 30643264

[62] Mukherjee N Corcoran DL Nusbaum JD Reid DW Georgiev S Hafner M. Integrative regulatory mapping indicates that the RNA-binding protein HuR couples pre-mRNA processing and mRNA stability. Mol Cell. (2011) 43:327–39. doi: 10.1016/j.molcel.2011.06.007 PMC3220597 21723170

[63] Noh JH Kim KM Abdelmohsen K Yoon JH Panda AC Munk R. HuR and GRSF1 modulate the nuclear export and mitochondrial localization of the lncRNA RMRP. Genes Dev. (2016) 30:1224–39. doi: 10.1101/gad.276022.115 PMC4888842 27198227

[64] Palanisamy K Tsai TH Yu TM Sun KT Yu SH Lin FY. RNA-binding protein, human antigen R regulates hypoxia-induced autophagy by targeting ATG7/ATG16L1 expressions and autophagosome formation. J Cell Physiol. (2019) 234:7448–58. doi: 10.1002/jcp.27502 30317574

[65] Li XX Xiao L Chung HK Ma XX Liu X Song JL. Interaction between huR and circPABPN1 modulates autophagy in the intestinal epithelium by altering ATG16L1 translation. Mol Cell Biol. (2020) 40(6):e00492-19. doi: 10.1128/MCB.00492-19 31932481 PMC7048268

[66] Zhao J Zhao Z Ying P Zhou Y Xu Z Wang H. METTL3-mediated m(6) A modification of circPRKAR1B promotes Crohn’s colitis by inducing pyroptosis via autophagy inhibition. Clin Trans med. (2023) 13:e1405. doi: 10.1002/ctm2.1405 PMC10485333 37679886

[67] Chen Y Lin Y Shu Y He J Gao W. Interaction between N(6)-methyladenosine (m(6)A) modification and noncoding RNAs in cancer. Mol cancer. (2020) 19:94. doi: 10.1186/s12943-020-01207-4 32443966 PMC7243333

[68] Liu J Yue Y Han D Wang X Fu Y Zhang L. A METTL3-METTL14 complex mediates mammalian nuclear RNA N6-adenosine methylation. Nat Chem Biol. (2014) 10:93–5. doi: 10.1038/nchembio.1432 PMC3911877 24316715

[69] Yang L Wu G Wu Q Peng L Yuan L. METTL3 overexpression aggravates LPS-induced cellular inflammation in mouse intestinal epithelial cells and DSS-induced IBD in mice. Cell Death discov. (2022) 8:62. doi: 10.1038/s41420-022-00849-1 35165276 PMC8844074

[70] Yang Y Hsu PJ Chen YS Yang YG. Dynamic transcriptomic m(6)A decoration: writers, erasers, readers and functions in RNA metabolism. Cell Res. (2018) 28:616–24. doi: 10.1038/s41422-018-0040-8 PMC5993786 29789545

[71] Yoo JH Holubar S Rieder F. Fibrostenotic strictures in Crohn’s disease. Intest Res. (2020) 18:379–401. doi: 10.5217/ir.2019.09148 32259917 PMC7609387

[72] Cherubini A Barilani M Rossi RL Jalal MMK Rusconi F Buono G. FOXP1 circular RNA sustains mesenchymal stem cell identity via microRNA inhibition. Nucleic Acids Res. (2019) 47:5325–40. doi: 10.1093/nar/gkz199 PMC6547427 30937446

[73] Yin J Ye YL Hu T Xu LJ Zhang LP Ji RN. Hsa_circRNA_102610 upregulation in Crohn’s disease promotes transforming growth factor-β1-induced epithelial-mesenchymal transition via sponging of hsa-miR-130a-3p. World J gastroenterol. (2020) 26:3034–55. doi: 10.3748/wjg.v26.i22.3034 PMC7304108 32587447

[74] Shah SC Itzkowitz SH. Colorectal cancer in inflammatory bowel disease: mechanisms and management. Gastroenterology. (2022) 162:715–30.e3. doi: 10.1053/j.gastro.2021.10.035 34757143 PMC9003896

[75] Yuan G Chen T Zhang H Cao Q Qiu Y Que B. Comprehensive analysis of differential circular RNA expression in a mouse model of colitis-induced colon carcinoma. Mol carcinogen. (2018) 57:1825–34. doi: 10.1002/mc.v57.12 30182433

[76] Zeng X Tang J Zhang Q Wang C Qi J Wei Y. CircHIPK2 contributes cell growth in intestinal epithelial of colitis and colorectal cancer through promoting TAZ translation. Adv Sci (Weinheim Baden-Wurttemberg Germany). (2024) 11:e2401588. doi: 10.1002/advs.202401588 PMC11425914 38981023

[77] Wan D Wang S Xu Z Zan X Liu F Han Y. PRKAR2A-derived circular RNAs promote the Malignant transformation of colitis and distinguish patients with colitis-associated colorectal cancer. Clin Trans med. (2022) 12:e683. doi: 10.1002/ctm2.v12.2 PMC8858608 35184406

[78] Du WW Zhou C Yang H Wen S Chen Y Chen EX. Aggravated Ulcerative Colitis via circNlgn-Mediated Suppression of Nuclear Actin Polymerization. Res (Washington DC). (2024) 7:0441. doi: 10.34133/research.0441 PMC11342054 39183944

[79] Yuan YY Wu H Chen QY Fan H Shuai B. Construction of the underlying circRNA-miRNA-mRNA regulatory network and a new diagnostic model in ulcerative colitis by bioinformatics analysis. World J Clin cases. (2024) 12:1606–21. doi: 10.12998/wjcc.v12.i9.1606 PMC10989427 38576737

[80] An Q Yang S Tao J Yang M Ma Z Gao Q. CIRC_0085323 SILENCING INHIBITS TNF-A-INDUCED NORMAL HUMAN COLONIC EPITHELIAL CELL INFLAMMATION AND APOPTOSIS THROUGH THE MIR-495-3P/TRAF3 AXIS. Shock (Augusta Ga). (2023) 60:298–305. doi: 10.1097/SHK.0000000000002167 37606890

[81] Meganck RM Borchardt EK Castellanos Rivera RM Scalabrino ML Wilusz JE Marzluff WF. Tissue-dependent expression and translation of circular RNAs with recombinant AAV vectors *in vivo* . Mol Ther Nucleic Acids. (2018) 13:89–98. doi: 10.1016/j.omtn.2018.08.008 30245471 PMC6154398

[82] Armstrong AW Read C. Pathophysiology, clinical presentation, and treatment of psoriasis: A review. Jama. (2020) 323:1945–60. doi: 10.1001/jama.2020.4006 32427307

[83] He AT Liu J Li F Yang BB. Targeting circular RNAs as a therapeutic approach: current strategies and challenges. Signal transduct target Ther. (2021) 6:185. doi: 10.1038/s41392-021-00569-5 34016945 PMC8137869

[84] Cui X Bao L Wang X Chen C. The nano-intestine interaction: understanding the location-oriented effects of engineered nanomaterials in the intestine. Small (Weinheim an der Bergstrasse Germany). (2020) 16:e1907665. doi: 10.1002/smll.201907665 32347646

[85] Shi X Wang B Feng X Xu Y Lu K Sun M. circRNAs and exosomes: A mysterious frontier for human cancer. Mol Ther Nucleic Acids. (2020) 19:384–92. doi: 10.1016/j.omtn.2019.11.023 PMC6939016 31887549

[86] Guo JU Agarwal V Guo H Bartel DP. Expanded identification and characterization of mammalian circular RNAs. Genome Biol. (2014) 15:409. doi: 10.1186/s13059-014-0409-z 25070500 PMC4165365

[87] Jeck WR Sorrentino JA Wang K Slevin MK Burd CE Liu J. Circular RNAs are abundant, conserved, and associated with ALU repeats. RNA (New York NY). (2013) 19:141–57. doi: 10.1261/rna.035667.112 PMC3543092 23249747

[88] Wesselhoeft RA Kowalski PS Anderson DG. Engineering circular RNA for potent and stable translation in eukaryotic cells. Nat commun. (2018) 9:2629. doi: 10.1038/s41467-018-05096-6 29980667 PMC6035260

[89] Huang S Li X Zheng H Si X Li B Wei G. Loss of super-enhancer-regulated circRNA nfix induces cardiac regeneration after myocardial infarction in adult mice. Circulation. (2019) 139:2857–76. doi: 10.1161/CIRCULATIONAHA.118.038361 PMC6629176 30947518

[90] Toro N Mestre MR Martínez-Abarca F González-Delgado A. Recruitment of reverse transcriptase-cas1 fusion proteins by type VI-A CRISPR-cas systems. Front microbiol. (2019) 10:2160. doi: 10.3389/fmicb.2019.02160 31572350 PMC6753606

